# Waning of specific antibodies against Delta and Omicron variants five months after a third dose of BNT162b2 SARS-CoV-2 vaccine in elderly individuals

**DOI:** 10.3389/fimmu.2022.1031852

**Published:** 2022-11-14

**Authors:** Yun Shan Goh, Angeline Rouers, Siew-Wai Fong, Nicole Ziyi Zhuo, Pei Xiang Hor, Chiew Yee Loh, Yuling Huang, Vanessa Kexin Neo, Isaac Kai Jie Kam, Bei Wang, Eve Zi Xian Ngoh, Siti Nazihah Mohd Salleh, Raphael Tze Chuen Lee, Surinder Pada, Louisa Jin Sun, Desmond Luan Seng Ong, Jyoti Somani, Eng Sing Lee, NCID Study Group, Jocelyn Jin Yu, COVID-19 Study Group, Adeline C.Y. Chua, Sebastian Maurer-Stroh, Cheng-I Wang, Yee‐Sin Leo, Ee Chee Ren, David C. Lye, Barnaby Edward Young, Lisa F. P. Ng, Laurent Renia

**Affiliations:** ^1^ ASTAR Infectious Diseases Labs (ASTAR ID Labs), Agency for Science, Technology and Research (ASTAR), Singapore, Singapore; ^2^ Singapore Immunology Network, Agency for Science, Technology and Research (A*STAR), Singapore, Singapore; ^3^ Bioinformatics Institute, Agency for Science, Technology and Research (A*STAR), Singapore, Singapore; ^4^ Global Data Science Initiative (GISAID), Munich, Germany; ^5^ Infectious Diseases, Ng Teng Fong General Hospital, Singapore, Singapore; ^6^ Infectious Diseases, Alexandra Hospital, Singapore, Singapore; ^7^ National University Polyclinic, Singapore, Singapore; ^8^ Division of Infectious Diseases, Department of Medicine, National University Hospital, National University Health System, Singapore, Singapore; ^9^ Clinical Research Unit, National Healthcare Group Polyclincs, Singapore, Singapore; ^10^ Lee Kong Chian School of Medicine, Nanyang Technological University, Singapore, Singapore; ^11^ National Public Health Laboratory, National Centre for Infectious Diseases (NCID), Singapore, Singapore; ^12^ Department of Biological Sciences, National University of Singapore, Singapore, Singapore; ^13^ National Centre for Infectious Diseases (NCID), Singapore, Singapore; ^14^ Department of Infectious Diseases, Tan Tock Seng Hospital, Singapore, Singapore; ^15^ Saw Swee Hock School of Public Health, National University of Singapore, Singapore, Singapore; ^16^ Department of Medicine, Yong Loo Lin School of Medicine, National University of Singapore, Singapore, Singapore; ^17^ Department of Microbiology and Immunology, Yong Loo Lin School of Medicine, National University of Singapore, Singapore, Singapore; ^18^ National Institute of Health Research, Health Protection Research Unit in Emerging and Zoonotic Infections, University of Liverpool, Liverpool, United Kingdom; ^19^ Institute of Infection, Veterinary and Ecological Sciences, University of Liverpool, Liverpool, United Kingdom; ^20^ School of Biological Sciences, Nanyang Technological University, Singapore, Singapore

**Keywords:** SARS-CoV-2, COVID-19, S protein, antibodies, T cells, variant, mRNA vaccine, booster

## Abstract

The emergence of new SARS-CoV-2 variants, such as the more transmissible Delta and Omicron variants, has raised concerns on efficacy of the COVID-19 vaccines. Here, we examined the waning of antibody responses against different variants following primary and booster vaccination. We found that antibody responses against variants were low following primary vaccination. The antibody response against Omicron was almost non-existent. Efficient boosting of antibody response against all variants, including Omicron, was observed following a third dose. The antibody response against the variants tested was significantly higher at one month following booster vaccination, compared with two months following primary vaccination, for all individuals, including the low antibody responders identified at two months following primary vaccination. The antibody response, for all variants tested, was significantly higher at four months post booster than at five months post primary vaccination, and the proportion of low responders remained low (6-11%). However, there was significant waning of antibody response in more than 95% of individuals at four months, compared to one month following booster. We also observed a robust memory B cell response following booster, which remained higher at four months post booster than prior to booster. However, the memory B cell responses were on the decline for 50% of individuals at four months following booster. Similarly, while the T cell response is sustained, at cohort level, at four months post booster, a substantial proportion of individuals (18.8 – 53.8%) exhibited T cell response at four months post booster that has waned to levels below their corresponding levels before booster. The findings show an efficient induction of immune response against SARS-CoV-2 variants following booster vaccination. However, the induced immunity by the third BNT162b2 vaccine dose was transient. The findings suggest that elderly individuals may require a fourth dose to provide protection against SARS-CoV-2.

## Introduction

The Pfizer/BioNTech BNT162b2 mRNA vaccine, one of the most administered worldwide, is a two-dose regimen administered 21 days apart as the primary vaccination series. It has demonstrated 65% and 90% vaccine efficacy against infection and severe disease respectively (1). However, along with many others (2–4), we have reported waning of antibody response at six months post BNT162b2 primary vaccination (5). This is of concern as many new SARS-CoV-2 variants, such as the Delta and Omicron variants, have emerged with multiple mutations that may increase disease severity, transmissibility, and immune evasion. A third BNT162b2 dose boosted antibody responses and demonstrated 95% vaccine efficacy against severe disease (6). Real-world immunogenicity data describing the antibody kinetics following booster vaccination are not yet available, especially in the elderly who are a high disease risk group. Here, we evaluated the immune response in elderly at two and five months following primary vaccination, and one and four months following a third dose.

## Methods

### Ethics statement and study population

The study design and protocol for the COVID-19 PROTECT study group were assessed by National Healthcare Group (NHG) Domain Specific Review Board (DSRB) and approved under study number 2012/00917. Written informed consent was obtained in accordance with the Declaration of Helsinki for Human Research.

A cohort of 36 individuals (**Supplementary Table 1**), aged 61-81 (median age = 71), were recruited. Two doses of Pfizer/BioNTech BNT162b2 mRNA primary vaccination were administered 21 days apart. Blood samples were collected at two months following primary vaccination (T1), five months following primary vaccination (T2), and one month (T3) and four months (T4) following a third dose (administered 189-270 days post first-dose). All individuals in the study are negative for antibodies against the nucleocapsid protein of SARS-CoV-2.

### Spike protein flow cytometry-based assay (SFB assay) for antibody detection

The SFB assay was performed as previously described (7, 8). The pTT5LnX-CoV-SP (expressing SARS-CoV-2 Spike protein, Genbank: YP_009724390.1) was used as a template plasmid to generate Spike gene of Alpha (B.1.1.7), Beta (B.1.351), Gamma (P.1), Kappa (B.1.617.1), Delta (B.1.617.2), Delta Plus (B.1.617.2, AY.2), and Omicron (B.1.1.529) using QuickChange Lightning Multi Site-Directed Mutagenesis Kit (Agilent). Cells, expressing the spike protein of either WT or variant, were seeded at 1.5 x 10^5^ cells/well in 96 well V-bottom plates. Cells were incubated with human serum (diluted 1:100 in 10% FBS) followed by a secondary incubation with a double stain, comprising Alexa Fluor 647-conjugated anti-human IgG (1:500 dilution) and propidium iodide (PI; 1:2500 dilution). Cells were acquired using a BD Biosciences LSR4 laser and analyzed using FlowJo (Tree Star). Gating strategy is described in **Supplementary Figure 1**. The assay was performed as two independent experiments, each with technical duplicates. Expression of the different spike proteins on the cell surface was confirmed by ACE-2-HuFc binding (**Supplementary Figure 2**). Low responders were arbitrarily defined as individuals with antibody response below 25^th^ percentile of the cohort response (n=36) at two months following primary vaccination (T1).

### Memory B cell ELISPOT

SARS-CoV-2 RBD-specific memory B cell numbers were counted using ELISpot. MultiScreenHTS IP Filter Plate, 0·45 µm plates (Merck Millipore) were coated overnight at 4°C with purified anti-human-IgG (MT91/145, Mabtech) or purified anti-human-IgA prepared at 15 μg/mL in PBS. Plates were washed and blocked for 30 min at room temperature with RPMI + 10% FBS. 1 x 10^6^ PBMCs were resuspended in 1 mL RPMI + 10% FBS + 1 μg/mL R848 + 10 ng/mL IL-2, and incubated at 37°C, 5% CO_2_ for 5 days to differentiate memory B cells into antibody-secreting cells. After incubation, cells were counted, and 100,000 or 400,000 live cells were taken for ELISpot plating to determine RBD-specific memory B cell numbers. Total IgG secreting cells were determined by plating 1500 or 3000 live cells. Cells were incubated for 18-22 h at 37°C, 5% CO_2_ in the ELISpot plate before detection. A combination of RBD-WASP/anti-WASP-ALP or anti-IgG-biotinylated/streptavidin-ALP (Mabtech) were used to detect RBD-specific or total IgG secreting cells respectively.

### IFN-γ/IL-2/IL-4/IL-5/IL-13 FluoroSpot assays

PBMCs were incubated overnight in RPMI-1640 + 10% Human AB Serum + 1% Penicillin Streptomycin + 1% 200g/mL D-glucose. FluoroSpot assays were used to measure CD8, CD4 Th1 and Th2 responses. PBMCs were stimulated in duplicates with WT SARS-CoV-2 spike peptide pool (5) (JPT Peptide Technologies) with 0.1μg/mL co-stimulator anti-CD28 (mAb CD28A). The peptide pool for CD8 FluoroSpot assay consists of 9-mers (n=211) while the peptide pool for CD4 FluoroSpot assay consists of 15-mers (n=315). CD8 and CD4 Th1 and CD4 Th2 responses were measured using Human IFN-γ/IL-2 FluoroSpot PLUS kits and custom Human IL-4/IL-5/IL-13 FluoroSpot FLEX kits respectively, following manufacturer’s instructions (MabTech).

In brief, for IFN-γ/IL-2 FluoroSpot assays, PVDF plates pre-coated with IFN-γ mAb (1-D1K) and IL-2 mAb (MT2A91/2C95) were washed with sterile phosphate buffered saline (PBS) and blocked with R10 medium. Following overnight incubation with the cells, plates were washed with PBS and incubated with detection antibodies anti-IFN-γ mAb (7-B6-1-BAM) and anti-IL2 mAb (MT8G10, biotinylated) diluted in PBS with 0.1% BSA. Plates were then washed with PBS and incubated with fluorophore conjugates for IFN-γ (anti-BAM-490) and IL-2 (SA-550) in PBS with 0.1% BSA. Plates were washed and incubated with ready-to-use fluorescent enhancer II.

For IL-4/IL-5/IL-13 FluoroSpot assays, the PVDF plates were activated with 15µL 35% EtOH per well. Plates were washed with cell culture water and incubated with IL-4 mAb (IL4-I), IL-5 mAb (TRFK5) and IL-13 mAb (MT1318) in PBS. After overnight incubation, plates were washed with sterile PBS and blocked with R10 medium. Following overnight incubation with the cells, plates were washed with PBS and incubated with detection antibodies anti-IL4 mAb (IL4-II), anti-IL5 mAb (5A10) and anti-IL13 mAb (25K2) diluted in PBS with 0.1% BSA. Plates were then washed with PBS and incubated with fluorophore conjugates for IL-4 (SA-550), IL-5 (anti-WASP-640) and IL-13 (anti-BAM-490) in PBS with 0.1% BSA. Plates were washed and incubated with ready-to-use fluorescent enhancer II for 15 minutes at RT.

For all FluoroSpot assays, plates were then emptied and dried overnight before analysis with Mabtech IRIS FluoroSpot and ELISpot reader using FITC filter for IFN-γ and IL-13, Cy3 filter for IL-2 and IL-4, Cy5 filter for IL-5. Spots were calculated based on the average of two wells using the MabTech IRIS Immunospot reader Apex software.

### Statistical analysis

Statistical analysis was performed using GraphPad Prism 7. To compare between multiple groups, Kruskal-Wallis tests and *post hoc* tests using Dunn’s multiple comparison tests were used. To compare between timepoints, Friedman tests and *post hoc* tests using Dunn’s multiple comparison tests were used. *p* < 0.05 was considered statistically significant. To analyze the antibody response and cellular response against WT Spike at T3 (upper panel) and T4 (lower panel) for correlation with age, spearman correlation was used.

## Results

In this study, we followed a group of elderly individuals (median age = 71), n=36, who received two doses of Pfizer/BioNTech BNT162b2 mRNA primary vaccination administered 21 days apart, and a third dose administered 189-270 days post first-dose. The cohort is part of a bigger cohort of 312 individuals (5) who received the primary vaccination consisting of two doses of Pfizer/BioNTech BNT162b2 mRNA doses in January – May 2021, administered 21 days apart. The individuals were grouped by age: (1) young, aged < 60 years old, and (2) elderly, aged ≥ 60 years old. In August 2021, in view of the emerging variants, Singapore has taken up a staggered approach to give priority to elderly individuals and has recommended for the elderly to receive a booster dose five months following their primary vaccination. Younger individuals, aged > 30 years old, were offered a booster dose in October 2021. In line with this approach and timeline, a group of 36 elderly individuals were randomly selected in August/September 2021 and offered a third dose five month following their primary vaccination. Analysis of samples were expanded to focus on variant-specific responses at two months following primary vaccination (T1), five months following primary vaccination (T2), and one month (T3) following the third dose, and more importantly, to investigate the longevity of the response at four months (T4) following the third dose.

**Figure 1:**
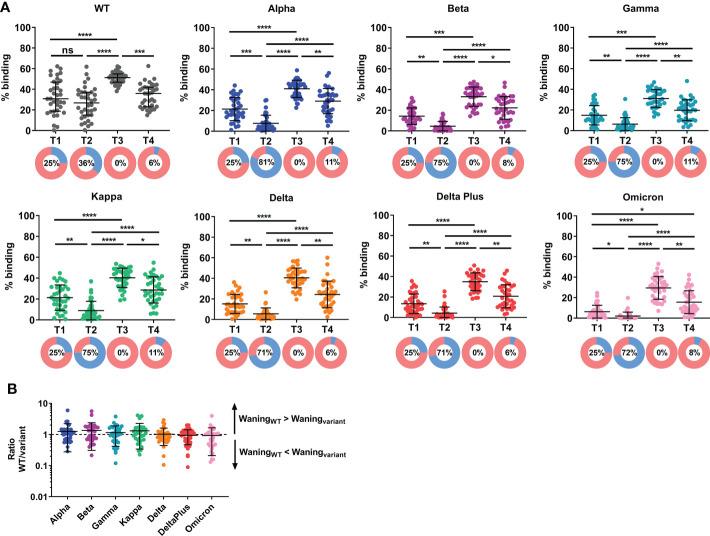
Induction and waning of antibody response against WT and variant Spike following booster vaccination. Plasma samples were screened for binding against full-length **(A)** wildtype (WT) and variant Spike (Alpha, Beta, Gamma, Kappa, Delta, Delta Plus and Omicron) in elderly individuals at two months (T1) and five months (T2) following primary vaccination, and one month (T3) and four months (T4) following a third dose (administered 189-270 days post first-dose). N=36. Pie chart shows the proportion of individuals with antibody responses above 25^th^ percentile of cohort response at two months following primary vaccination, T1 (in pink) and individuals with antibody responses below 25^th^ percentile of cohort response at T1 (in blue; defined as low responders); Number in pie chart indicates the proportion of low responders (blue in pie chart). **(B)** The reduction in antibody response against variant Spike at T4 (compared with T3) was compared with the reduction in WT Spike-specific and expressed as a ratio (WT/variant). A ratio >1 indicates that the waning of antibody response against WT Spike is more than waning of antibody response against variant Spike. N=36. To compare between multiple groups, Kruskal-Wallis tests and *post hoc* tests using Dunn’s multiple comparison tests were used. *, P-value <0.05; **. P-value <0.01; ***, P-value <0.001; ****, P-value <0.0001; ns, not significant.

At two months following primary vaccination (T1), there was an efficient induction of antibody response against WT Spike (**Figure 1**). However, there remained a substantial proportion of low responders, arbitrarily defined as individuals with antibody response below 25^th^ percentile of the cohort response at T1. We previously found that the IgG response against the WT and variant Spike strongly correlated with the capability to inhibit pseudovirus and live virus neutralization expressing the corresponding Spike (9, 10). Here, we extended the antibody analysis to examine variant-specific responses. The IgG antibody response against variant Spike was lower than against WT Spike at all time-points examined (**Supplementary Figure 3**). The lower variant Spike binding efficacy, compared to WT, by the plasma samples is unlikely due to lower Spike expression as we have found higher ACE-2 binding efficacy with the variants, compared to WT (**Supplementary Figure 2**). More notably, antibody response against Omicron was the lowest – it was negligible, even at just two months following primary vaccination. We did not observe a significant decrease in specific antibody response against WT full-length Spike at five months (T2) (compared to two months, T1) post primary vaccination. However, the antibody response against Spike decreased for all variants examined, including Alpha, Beta, Gamma, Kappa, Delta, Delta Plus, and Omicron (**Figure 1A**) at T2. The proportion of low responders increased substantially, especially for antibody response against the variants (from 25% to 71-81%). Following the booster vaccination (third dose, T3), the IgG antibody response against WT and the variants examined was efficiently boosted, including all low responders. At four months post booster dose (T4), there was significant waning of antibody response (**Figure 1A**) against WT Spike in 35/36 (97.2%) of the individuals and against all variant Spike tested in 34/36 (94.4%) of the individuals, compared with the corresponding responses at one month post booster (T3). Despite the antibody waning, antibody responses at four months post booster dose were higher than that at five months post primary vaccination (**Figure 1A**). In fact, all individuals had a higher anti-S response at T4 than at T2, and this was consistent across responses against WT and the variants examined. The proportion of antibody low responders (those below 25^th^ percentile of the cohort response at T1) remained low (6-11%). The reduction in antibody response against variant Spike at T4 (compared with T3) was compared with the reduction in WT Spike-specific and expressed as a ratio (WT/variant; **Figure 1B**). A ratio >1 indicates that the waning of antibody response against WT Spike is more than waning of antibody response against variant Spike. We found no significant difference in the waning of variant Spike-specific antibody response, suggesting that antibody response against variant Spike wanes in a similar manner at four months post booster vaccination, for the variants examined.

**Figure 2:**
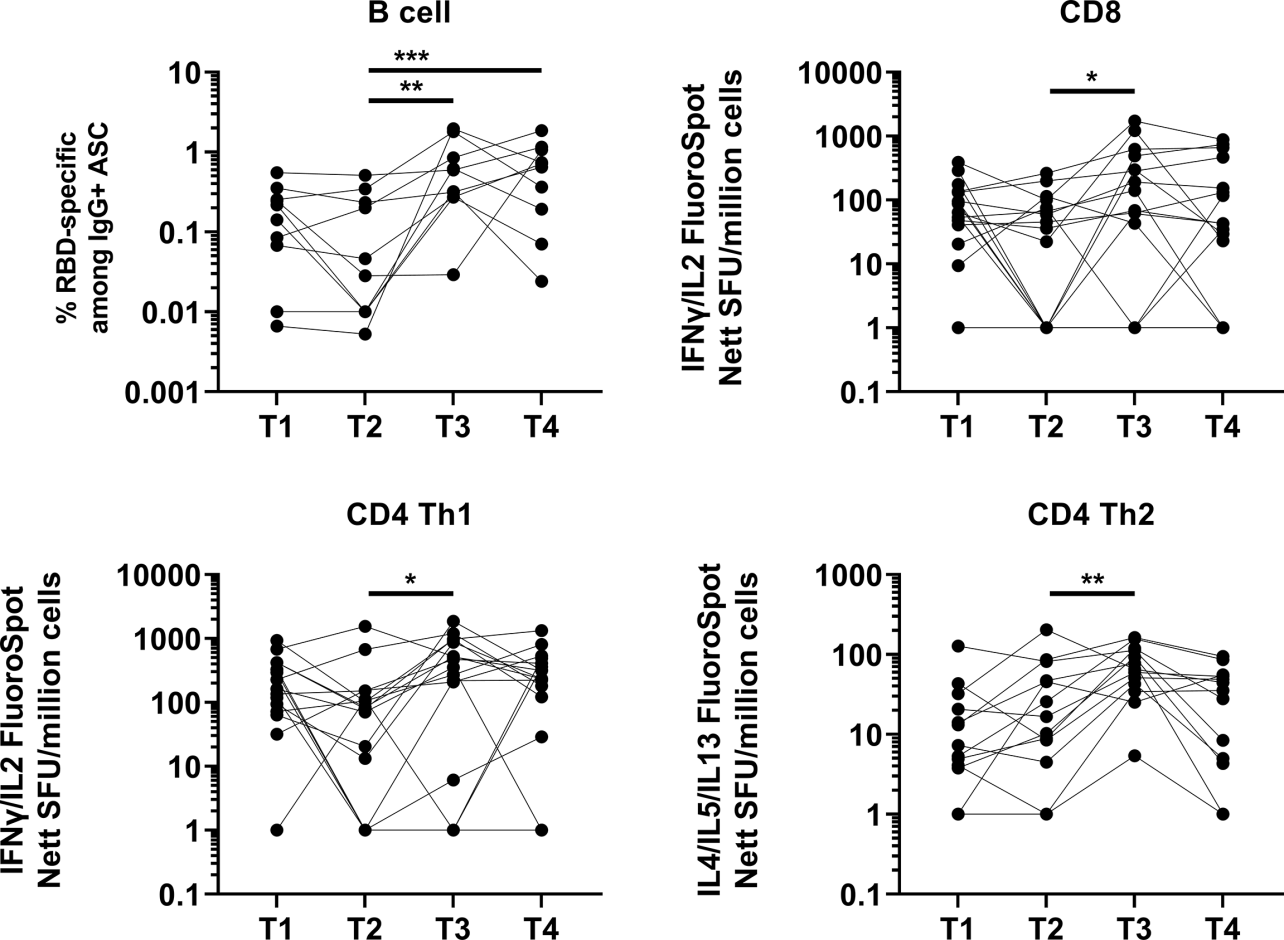
Induction and waning of memory B and T cell response against Spike following booster vaccination. RBD-specific memory B (N=10), Spike-protein-specific CD8 (N=16), CD4 Th1 (N=16) or CD4 Th2 (N=13) responses. For memory B cell response, frequency of IgG RBD-specific memory B cells among total IgG antibody secreting cells (ASCs) is presented. For CD8, CD4 Th1, and CD4 Th2 responses, data presented are spot forming units (SFU) per million PBMC from paired samples at four time-points. Each data point represents the normalized mean spot count from duplicate wells, after subtraction of medium-only control. To compare between timepoints, Friedman tests and *post hoc* tests using Dunn’s multiple comparison tests were used. *, P-value <0.05; **. P-value <0.01; ***, P-value <0.001.

We also analyzed the memory B and T cell response for a subset of randomly selected age-matched elderly individuals due to cell availability. The memory B cell response was efficiently induced in all tested individuals (10/10) at one month post booster vaccination and was not significantly lower at four months post booster vaccination (**Figure 2**). However, we observed a decrease in memory B cell response in 5/10 individuals (50%). In agreement with the antibody data, the memory B cell response at four months post booster vaccination remained higher than that observed before the booster (five months post primary vaccination). We showed that the T cell response, as assessed by ELISPOT, remained robust, with no significant difference in T cell response at five months post primary vaccination (**Figure 2**), compared with two months post primary vaccination. There was a significant increase in CD4 Th1 T cell response at one month following the booster dose (**Figure 2**). A total of 2/16 (12.5%) and 1/16 (6.25%) individuals did not develop a CD8 and CD4 Th1 T cell response following booster. While there was no significant difference in the T cell response at four months following the booster (compared to one month following booster) at the cohort level, we observed waning T cell response at four months following the booster (compared to one month following booster) in 8/16 (50%), 7/16 (43.8%) and 11/13 (84.6%) individuals for CD8, CD4 Th1 and CD4 Th2 T cell responses respectively. Importantly, 4/16 (25%), 3/16 (18.8%) and 7/13 (53.8%) had a lower CD8, CD4 Th1 and CD4 Th2 T cell response at four months following the booster (compared to five month post primary vaccination, before booster) respectively.

## Discussion

This study demonstrates the importance of the third dose for elderly individuals. While studies (2, 3, 5, 11, 12) have reported a negative correlation between age and antibody levels and also antibody waning months following vaccination, most are population-based. In this study focused on the elderly, we found that the antibody response against the variants, and more strikingly, against Omicron was low and almost non-existent following primary vaccination series, and a third dose was essential for induction of Omicron antibody response. In fact, the antibody response against the variants tested was significantly higher at one month following booster vaccination, compared with two months following primary vaccination. All, including the low responders, have a robust antibody response following the booster vaccination. Despite waning, the antibody response, for all variants tested, was still significantly higher at four months post booster than that at five months post primary vaccination. Most have robust antibody response, with the proportion of low responders remaining low (6-11%). We also observed a robust memory B cell response following booster vaccination, which remained higher than that observed prior to booster vaccination (at five months post primary vaccination). In addition to antibody, T cells are also important in the control of SARS-CoV-2 infections (13–15), where high levels of effector molecules by CD8 T cells in acute COVID-19 are associated with mild disease. We found robust T cell response at four months post booster vaccination at a cohort level. The efficacy of the fourth dose against infections has been modest in cohort study examining younger (16), and older age groups (17). Our findings suggest that the modest benefit of the fourth dose in the elderly may, in part, be due to a robust antibody response against the variants, even at four months following the third dose. Although waning of immune responses was observed, the decrease in antibody response at five months post booster vaccination (compared with one month post booster vaccination) did not correlate with a decrease in memory B cell response nor with a decrease in memory T cell response (**Supplementary Figure 4**). This suggests that, while the antibody responses may have waned for some of the individuals, the sustained memory response can still be triggered to induce protection. However, it is important to highlight that the memory B cell responses were on the decline for 50% of the individuals. Similarly, there were individuals who did not mount an increase in cellular response after the third dose, and a substantial proportion of individuals exhibited a waning T cell response at four months post booster, with responses below their corresponding levels before booster (five month post primary vaccination). While age has a negative impact on responses induced by primary vaccination (5, 18–20), we did not find a correlation between the immune response induced by the booster vaccination and age within this cohort of elderly individuals (**Supplementary Figure 5**). This may be due to the smaller age range of 61-81 in these individuals. Markedly lower responses have been reported in individuals aged over 80 years old following primary BNT162b2 vaccination (19). Nevertheless, the findings suggest that elderly individuals display waning immune responses following the transient increase after booster vaccination and may require a fourth dose. Heterologous boosting has been shown to provide better antibody response (21–23) and the better immune response may also include memory B and T cell responses. Whether another mRNA vaccine, such as Moderna’s mRNA-1273, or a vaccine based on a different platform such as a protein-based NVX-CoV2373 (Novavax) can offer better protection against COVID-19 as a heterologous booster remains to be determined.

## Data availability statement

The original contributions presented in the study are included in the article/**Supplementary Material**. Further inquiries can be directed to the corresponding author.

## Ethics statement

The studies involving human participants were reviewed and approved by National Healthcare Group Domain Specific Review Board. The patients/participants provided their written informed consent to participate in this study.

## Team authors in NCID study group are listed as below.

**Jocelyn Jin Yu**, National Centre for Infectious Diseases, Singapore; **Zheng Kuang Soh**, National Centre for Infectious Diseases, Singapore; **Yi Qing Chin**, National Centre for Infectious Diseases, Singapore; **Jonathan Jordon Lim**, National Centre for Infectious Diseases, Singapore; **Juwinda Ongko**, National Centre for Infectious Diseases, Singapore; Eshele Anak Libau, National Centre for Infectious Diseases, Singapore; **Mohammed Ridzwan Bin Abdullah**, National Centre for Infectious Diseases, Singapore; **Shiau Hui Diong**, National Centre for Infectious Diseases, Singapore; **Jefanie Teo**, National Centre for Infectious Diseases, Singapore; **He Ping Yeo**, National Centre for Infectious Diseases, Singapore.

## Team authors in COVID-19 study group are listed as below.

**Adeline C.Y. Chua**, A*STAR Infectious Diseases Labs, Agency for Science, Technology and Research; **Zi Wei Chang**, A*STAR Infectious Diseases Labs, Agency for Science, Technology and Research; **Samantha Y. T. Nguee**, A*STAR Infectious Diseases Labs, Agency for Science, Technology and Research; **Guillaume Carissimo**, A*STAR Infectious Diseases Labs, Agency for Science, Technology and Research; **Yong Jie Tan**, A*STAR Infectious Diseases Labs, Agency for Science, Technology and Research; **Joel Xu En Wong**, A*STAR Infectious Diseases Labs, Agency for Science, Technology and Research; **Anthony Torres-Ruesta**, A*STAR Infectious Diseases Labs, Agency for Science, Technology and Research; **Siti Naqiah Amrun**, A*STAR Infectious Diseases Labs, Agency for Science, Technology and Research; **Nicholas Kim-Wah Yeo**, A*STAR Infectious Diseases Labs, Agency for Science, Technology and Research; **Wendy Yehui Chen**, A*STAR Infectious Diseases Labs, Agency for Science, Technology and Research; **Alice Soh Meoy Ong**, A*STAR Infectious Diseases Labs, Agency for Science, Technology and Research.

## Author contributions

YG: conceptualized the study, designed and performed the experiments, analyzed data and wrote the manuscript. AR, S-WF, NZ, PH, CL, YH, VN, IK, BW, EN, SS, RL: designed and performed the experiments, analyzed data. SP, LS, DO, JS, EL: supervised and coordinated cohort recruitment and sample collection. NCID Study Group: cohort recruitment and sample collection. COVID-19 Cohort Study Group: processed samples. SM-S, CIW, LY-S, ER, DL, BY, LN, LR: conceptualized the study and reviewed the manuscript. All authors contributed to the article and approved the submitted version.

## Funding

This work was supported by the Biomedical Research Council (BMRC), A*CRUSE (Vaccine monitoring project), the A*ccelerate GAP-funded project (ACCL/19-GAP064-R20H-H) from Agency of Science, Technology and Research (A*STAR), Singapore National Medical Research Council COVID-19 Research Fund (COVID19RF-001; COVID19RF-007; COVID19RF-0008; COVID19RF-060), US Food and Drug Administration (#75F40120C00085) and A*STAR COVID-19 Research funding (H/20/04/g1/006).

## Conflict of interest

A patent application for the SFB assay has been filed Singapore patent #10202009679P: A Method Of Detecting Antibodies And Related Products.

The authors declare that the research was conducted in the absence of any commercial or financial relationships that could be construed as a potential conflict of interest.

## Publisher’s note

All claims expressed in this article are solely those of the authors and do not necessarily represent those of their affiliated organizations, or those of the publisher, the editors and the reviewers. Any product that may be evaluated in this article, or claim that may be made by its manufacturer, is not guaranteed or endorsed by the publisher.

## Supplementary material

Supplementary Figure 1 Spike binding to ACE-2. Binding was examined by SFB assay, where serially diluted ACE-2-HuFc was added to the cells, in place of plasma antibody. Bound ACE-2-HuFc were then detected by Alexa Fluor 647-conjugated anti-human IgG (1:500 dilution) and propidium iodide (PI; 1:2500 dilution).

Supplementary Figure 2 Variant Spike expression on cell surface. Expression was examined by SFB binding assay, where serially diluted ACE-2-HuFc was added to the cells, in place of plasma antibody. Bound ACE-2-HuFc were then detected by Alexa Fluor 647-conjugated anti-human IgG (1:500 dilution) and propidium iodide (PI; 1:2500 dilution). Cells were gated as described in **Supplementary Figure 1**.

Supplementary Figure 3 Antibody response against WT and variant Spike at the four time-points examined. Plasma samples (N=36) were screened for binding against full-length wildtype (WT) and variant Spike in elderly at two months (T1) and five months (T2) following primary vaccination, and one month (T3) and four months (T4) following a third dose (administered 189-270 days post first-dose).

Supplementary Figure 4 Correlation analysis between humoral and cellular responses. Difference in WT spike antibody response, T4 -T3, were analyzed for correlation with either difference in RBD-specific memory B cell response or Spike-specific CD8, CD4 Th1 or CD4 Th2 T cell responses. Spearman correlation analysis was used.

Supplementary Figure 5 Correlation analysis between immune response and age. Antibody response and cellular response against WT Spike at T3 (upper panel) and T4 (lower panel) were analyzed for correlation with age. Spearman correlation analysis was used.

Supplementary Table 1 Demographic information of study cohort.
