# Supplementary figures and images for: Waning of specific antibodies against Delta and Omicron variants five months after a third dose of BNT162b2 SARS-CoV-2 vaccine in elderly individuals
Source: Front Immunol. 2022 Nov 14;13:1031852. doi: 10.3389/fimmu.2022.1031852 (PMC9704817; doi:10.3389/fimmu.2022.1031852)

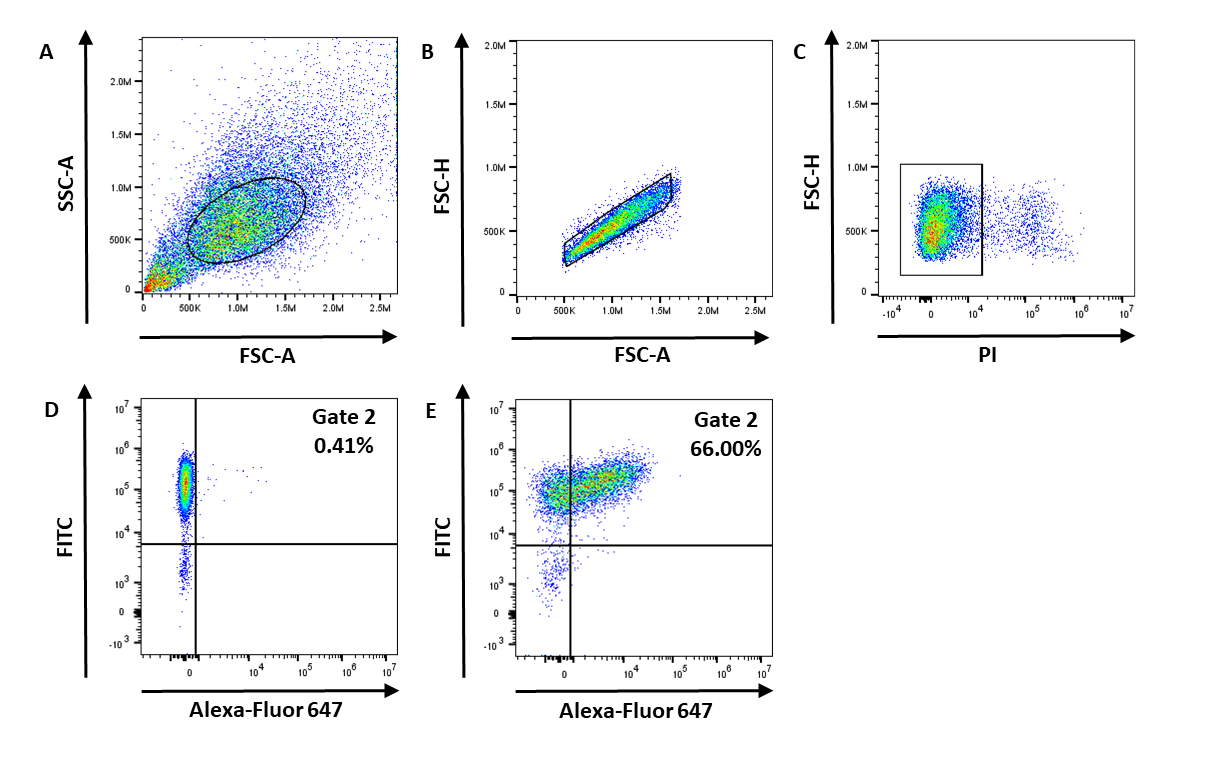

Supplement: Supplementary Figure 1 — Spike binding to ACE-2. Binding was examined by SFB assay, where serially diluted ACE-2-HuFc was added to the cells, in place of plasma antibody. Bound ACE-2-HuFc were then detected by Alexa Fluor 647-conjugated anti-human IgG (1:500 dilution) and propidium iodide (PI; 1:2500 dilution). [file Image_1.tif]

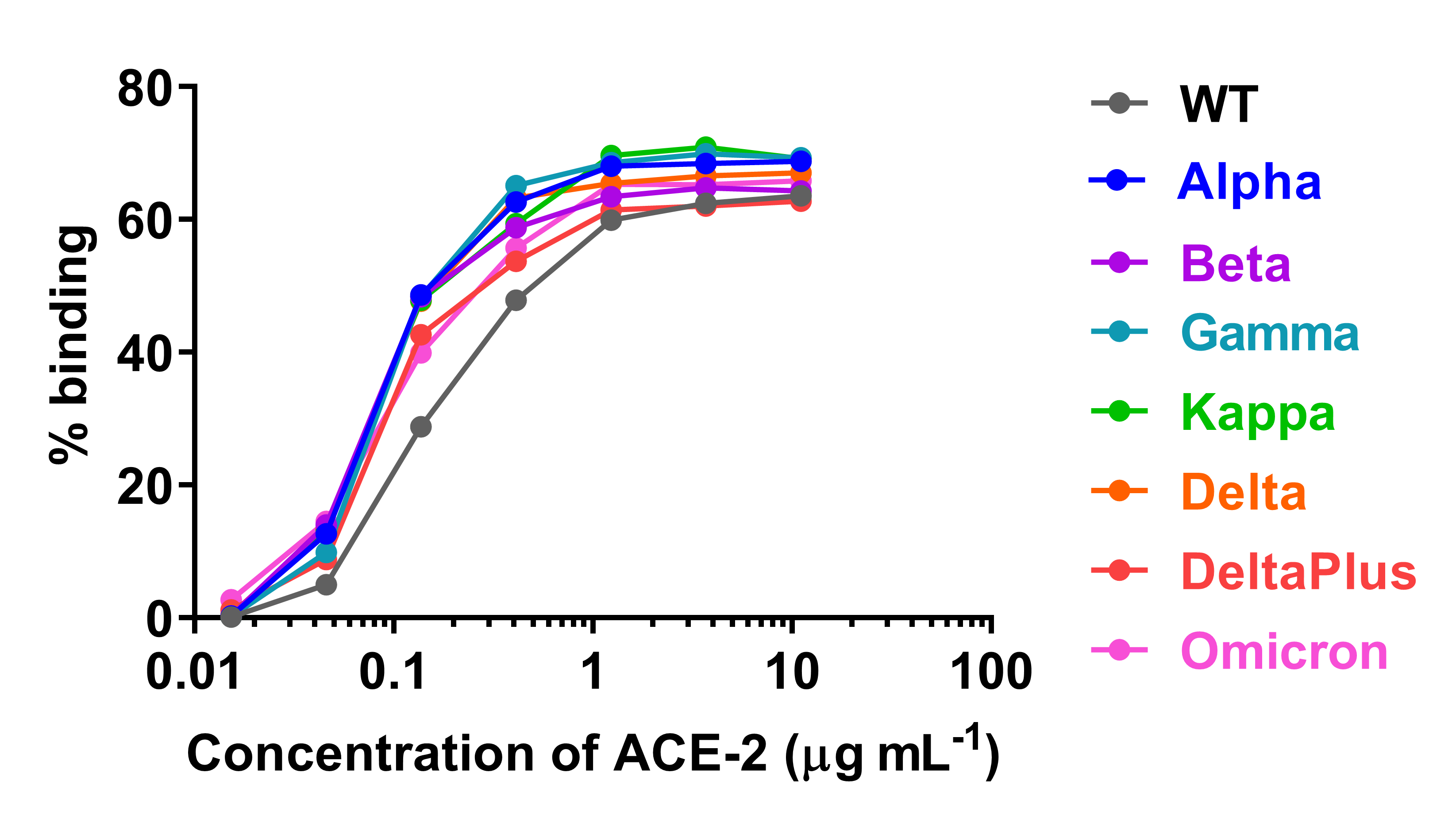

Supplement: Supplementary Figure 2 — Variant Spike expression on cell surface. Expression was examined by SFB binding assay, where serially diluted ACE-2-HuFc was added to the cells, in place of plasma antibody. Bound ACE-2-HuFc were then detected by Alexa Fluor 647-conjugated anti-human IgG (1:500 dilution) and propidium iodide (PI; 1:2500 dilution). Cells were gated as described in Supplementary Figure 1 . [file Image_2.tif]

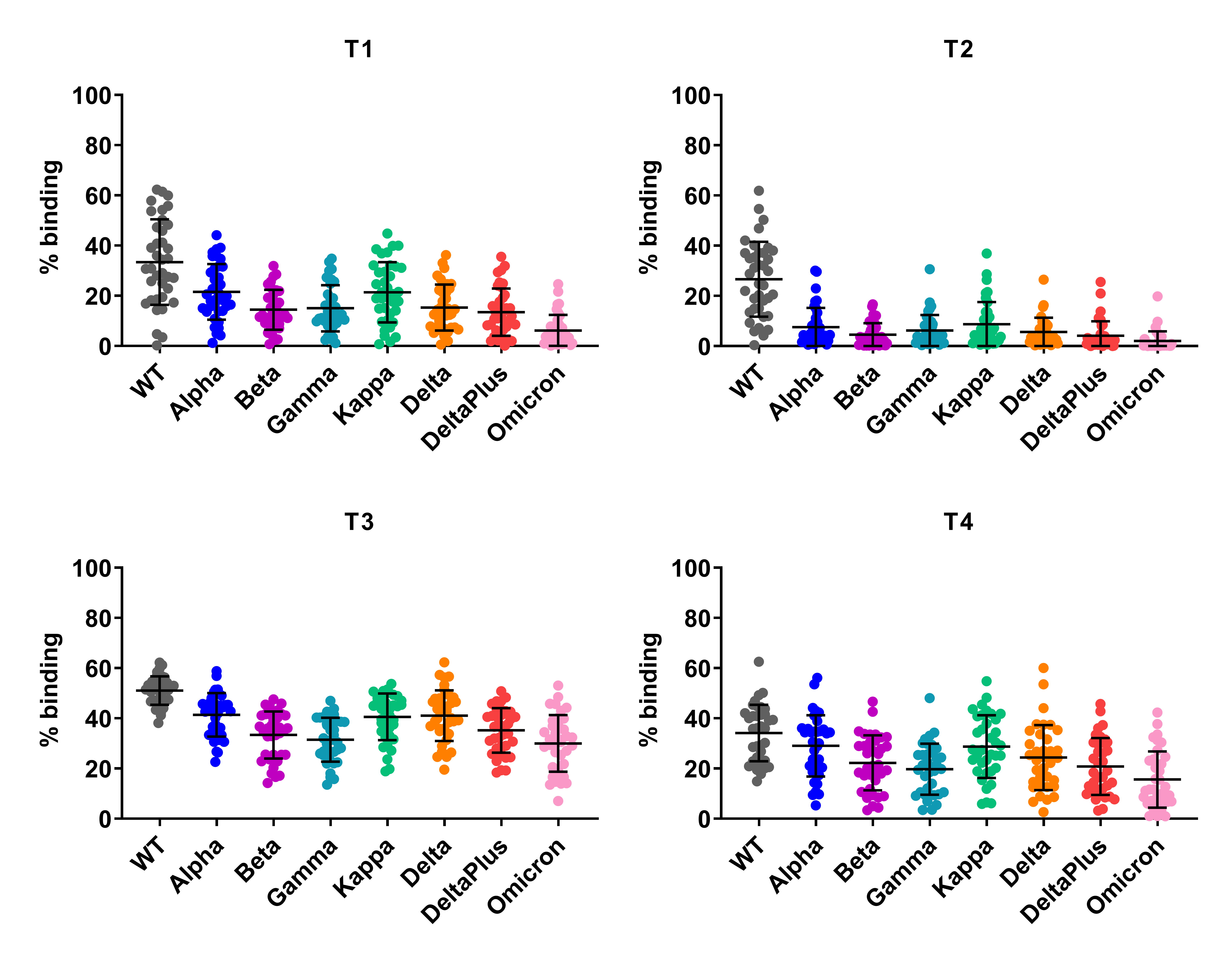

Supplement: Supplementary Figure 3 — Antibody response against WT and variant Spike at the four time-points examined. Plasma samples (N=36) were screened for binding against full-length wildtype (WT) and variant Spike in elderly at two months (T1) and five months (T2) following primary vaccination, and one month (T3) and four months (T4) following a third dose (administered 189-270 days post first-dose). [file Image_3.tif]

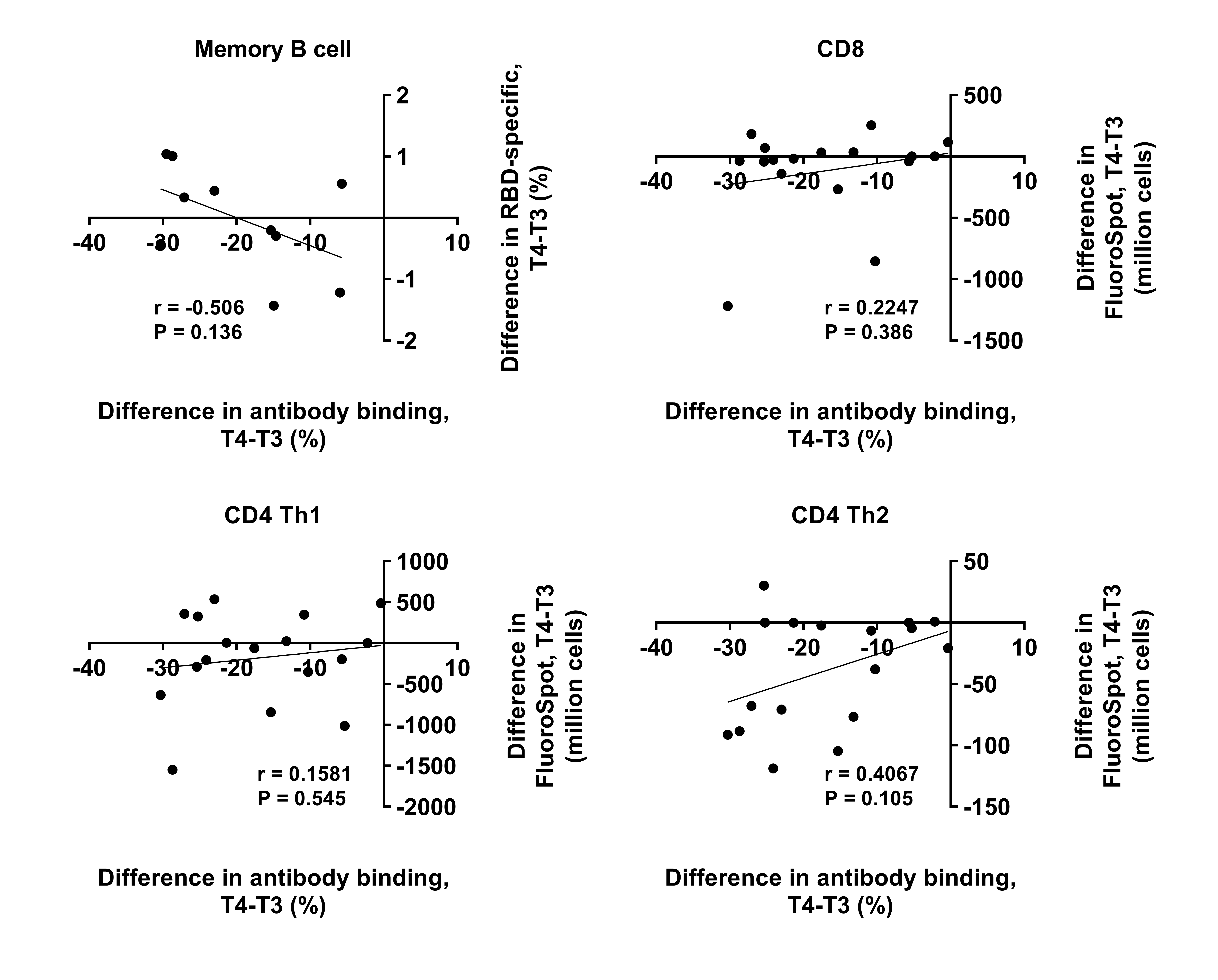

Supplement: Supplementary Figure 4 — Correlation analysis between humoral and cellular responses. Difference in WT spike antibody response, T4 -T3, were analyzed for correlation with either difference in RBD-specific memory B cell response or Spike-specific CD8, CD4 Th1 or CD4 Th2 T cell responses. Spearman correlation analysis was used. [file Image_4.tif]

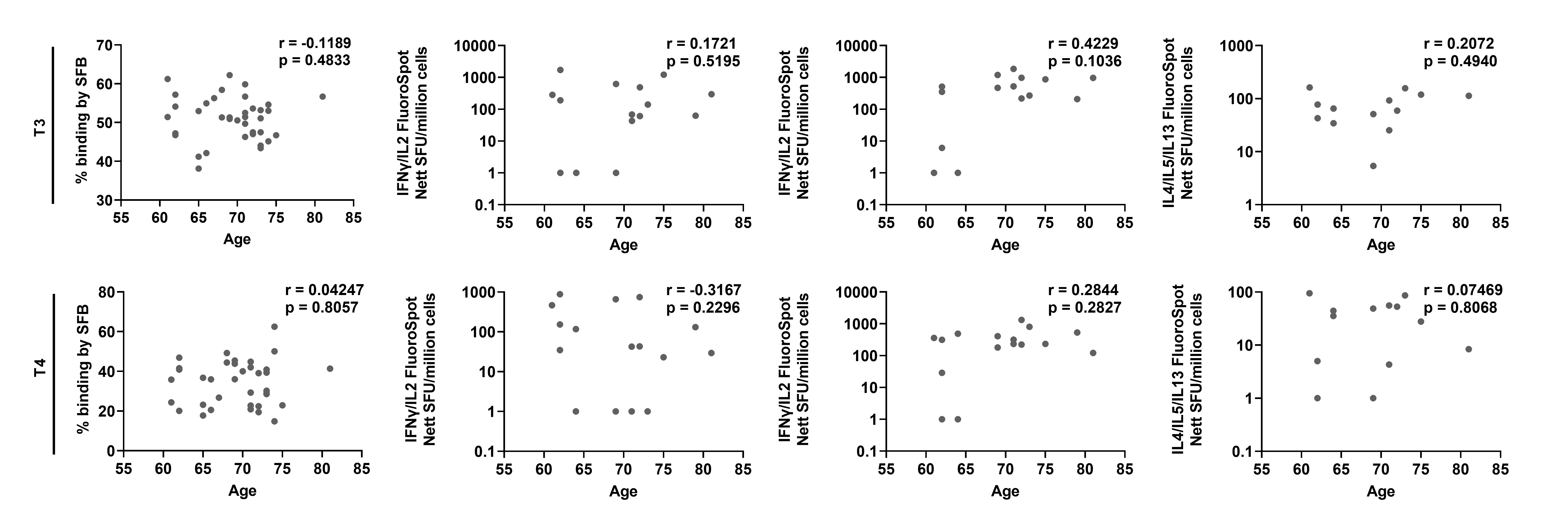

Supplement: Supplementary Figure 5 — Correlation analysis between immune response and age. Antibody response and cellular response against WT Spike at T3 (upper panel) and T4 (lower panel) were analyzed for correlation with age. Spearman correlation analysis was used. [file Image_5.tif]
