# Supplementary material for: Waning of specific antibodies against Delta and Omicron variants five months after a third dose of BNT162b2 SARS-CoV-2 vaccine in elderly individuals
Source: Front Immunol. 2022 Nov 14;13:1031852. doi: 10.3389/fimmu.2022.1031852 (PMC9704817; doi:10.3389/fimmu.2022.1031852)
Supplement: Supplementary Table 1 — Demographic information of study cohort. [file Table_1.docx]

**Supplementary Table 1. Demographic information of study cohort**

|  | N=36 |
| --- | --- |
| Age, median (range), years | 71 (61-81) |
| Gender, n (%) |  |
| Male | 26 (72.2) |
| Female | 10 (27.8) |
| Ethnicity, n (%) |  |
| Chinese | 29 (80.5) |
| Indians | 6 (16.6) |
| Malays | 1 (2.9) |
| Comorbidities, n (%) |  |
| Diabetes | 13 (36.0) |
| Hypertension | 22 (61.0) |
| Chronic lung diseases | 2 (5.6) |
| Chronic liver diseases | 1 (2.8) |
| Chronic renal diseases | 1 (2.8) |
| Organ transplantation | 0 (0.0.) |
| Autoimmune diseases | 0 (0.0) |
| Medications, n (%) |  |
| ^1^Antihypertensive | 20 (55.6) |
| ^2^Antidiabetic | 11 (30.6) |
| ^3^Anticholesterol | 19 (52.8) |
| ^4^Anti-inflammatory | 8 (22.3) |
| ^5^Antithrombotic | 4 (11.1) |
| ^6^COPD | 2 (5.6) |

^1^Includes Amlodipine, Valsartan, Bisoprolol, Atenolol, Verapramil, Coapprovel, Amlodipine, Losartan, Enalopril, Lisinopril, Telmistan, Irbesartan and Nifedipine. ^2^Includes Glipzide, Metformin, Empagliflozin, Dapagliflozin and Linagliptin. ^3^Includes Simvastatin, Ezetimise, Rosuvastatin Sandoz, Atorvastatin, Crestor, Lovastatin and Tenofibrate. ^4^Includes Colchicine, Non-steroidal anti-inflammatory drugs (NSAIDs) (Aspirin, Cardiprin), Corticosteroids (Prednisolone, Budesonide) and antihistamine (Loratadine). ^5^Includes Clopidogrel and Glyprin Asprin. ^5^Includes Salbutamol sulfate inhaler and Seletide. COPD: Chronic obstructive pulmonary disease.
